# Prenatal lead exposure: associations with growth and anthropometry in early childhood in a UK observational birth cohort study

**DOI:** 10.12688/wellcomeopenres.16338.2

**Published:** 2021-03-09

**Authors:** Caroline M. Taylor, Jean Golding, Katarzyna Kordas

**Affiliations:** 1 Centre for Academic Child Health, Bristol Medical School, University of Bristol, Bristol, Bristol, BS8 1NU, UK; 2 Department of Epidemiology and Environmental Health, School of Public Health and Health Professions, University at Buffalo, Buffalo, NY, USA

**Keywords:** ALSPAC, pregnancy, child, lead, growth, anthropometry

## Abstract

**Background:** Lead is a neurotoxic metal that crosses the placenta freely. It has adverse effects on a range of birth outcomes. The few studies reporting on the associations of prenatal exposure to lead and child growth have had conflicting results. This study aimed to examine the effect of prenatal exposure to lead on children’s growth from 4 to 61 months of age.

**Methods:** Pregnant women were enrolled in the UK Avon Longitudinal Study of Parents and Children (ALSPAC). Whole blood samples for pregnancies with a live birth were analysed for lead (n=4140). A 10% subsample of the offspring cohort (Children in Focus) were invited to clinics at 10 time points (4–61 months) at which anthropometric measurements were carried out; z-scores for height, weight and BMI were calculated using the 1990 British Growth Reference Standards. Associations between prenatal log _10_-lead concentrations and z-scores and other anthropometric measures were modelled using adjusted linear regression models in an imputed dataset for children who attended at least one clinic (n=574).

**Results:** The median prenatal blood lead concentration was 3.60 (IQR 2.61-4.16) µg/dl. There was no evidence for any associations of prenatal lead exposure with z-scores for BMI, height or weight in adjusted models from age 4 to 61 months. There were no associations for other anthropometric measures including mid-upper arm circumference, head circumference and waist circumference. There was some evidence for a weakly positive effect of prenatal lead exposure on head circumference in girls at age 43 and 61 months (at 61 months unstandardised B coefficient 1.59 (95% CI 0.12, 3.16) cm, p=0.048) but not at other ages.

**Conclusions:** There was no consistent evidence of associations between prenatal exposure to lead and measures of growth and anthropometry from age 4 to 61 months in this cohort of children in the UK.

## Introduction

Lead is a neurotoxic metal that is widespread in the environment. Many countries have adopted lead abatement measures in recent years, including the removal of lead from paint and petrol, resulting in declines in lead levels in children and adults ^1, 2^. Common sources of exposure still include water, diet, dust and soil ^3^, and smoking ^4^. Lead is still a public health concern, however, even in countries where exposure is relatively low: there is increasing awareness that adverse effects occur at all levels of lead exposure, with no lower limit for effects ^5– 7^.

Pregnancy is a critical time for exposure to lead for the mother and fetus. Once absorbed from the gastrointestinal tract or the respiratory system, lead is transported bound to erythrocytes and accumulates in bone (with a half-life of approximately 30 years). An increased demand for calcium means that maternal turnover of bone increases during pregnancy ^8^ and bone becomes the main source of lead in the blood ^9^. Lead crosses the placenta freely ^10, 11^ and can have adverse effects on a range of birth outcomes, including low birth weight and small-for-gestational-age, across a range of maternal blood lead levels ^7, 12– 14^. In accordance with the theory of the developmental origins of health and disease, constraints on fetal growth are likely to have a long-lasting effect on postnatal growth ^15^, and thus on child health and development. The potential mechanisms for the adverse effect of prenatal lead exposure on growth are largely undefined, but may include direct interference with bone cell function and/or disruption of hormones (such as vitamin D and thyroid hormones) that are involved in the synthesis of the bone matrix, as well as epigenetic mechanisms.

There have been conflicting results among the few studies that tested the associations of prenatal exposure to lead and subsequent measures of child growth in both cross-sectional ^16– 18^ and longitudinal ^19– 28^ analyses. Some have shown no associations despite relatively high ^16, 24, 26^ or likely high ^20^ maternal lead exposure; some have shown transient adverse effects in children up to about 15 months of age ^29^ or adverse effects in girls but not boys ^19, 23^. Others have suggested that prenatal lead exposure is associated with increased BMI in childhood ^17, 21, 25^. These varied findings likely reflect differences in exposure levels, the measurement tissue chosen (e.g. maternal blood, cord blood, maternal bone) and timing of sampling, differences in the length of time and the number of occasions that children are followed up, as well as children’s postnatal exposure to lead and other factors affecting growth.

To enable a clearer understanding of the association between prenatal lead exposure and growth trajectories, there is a need for further studies with longitudinal measures of growth. The Avon Longitudinal Study of Parents and Children (ALSPAC) is a birth cohort study in which prenatal lead exposure was measured as the mothers’ blood lead concentration (B-Pb) in the first trimester. The aim of this study was to evaluate associations of anthropometric measures in children aged between 4 and 61 months (z-scores for height, weight and BMI; head circumference, waist circumference and mid upper arm circumference (MUAC)) with a measure of prenatal lead exposure.

## Methods

### The ALSPAC study

The sample was derived from ALSPAC, a population-based study investigating environmental and genetic influences on the health, behaviour and development of children. All pregnant women in the former Avon Health Authority with an expected delivery date between 1 April 1991 and 31 December 1992 were eligible for the study; 14,541 pregnant women were enrolled, resulting in a cohort of 14,062 live births ^30, 31^. Further details of ALSPAC are available at www.bris.ac.uk/alspac and the study website contains details of all the data that are available through a fully searchable data dictionary and variable search tool ( http://www.bristol.ac.uk/alspac/researchers/our-data/). Ethical approval for the study was obtained from the ALSPAC Ethics and Law Committee and the Local Research Ethics Committee and conformed to the Declaration of Helsinki. Informed consent for the use of data collected via questionnaires and clinics was obtained from participants following the recommendations of the ALSPAC Ethics and Law Committee at the time.

### Exposure measurement: collection, storage and analysis of blood samples

Whole blood samples were collected in acid-washed vacutainers (Becton and Dickinson, Oxford, UK) by midwives who were collecting blood for clinical purposes with a vacutainer system. Samples were collected as early as possible in pregnancy (median of 11 weeks’ gestation (IQR 9–13 weeks) with a mode of 10 weeks). Whole blood samples were stored in the original tube at 4°C at the collection site before being transferred to the central Bristol laboratory within 1–4 days. Samples were kept at ambient temperature during transfer (up to 3 h). They were then stored at 4°C until analysis. Details of the analysis have been reported ^4, 32^. In brief, inductively coupled plasma mass spectrometry in standard mode (R. Jones, Centers for Disease Control and Prevention (CDC), Bethesda, MD, USA CDC; Method 3009.1) was used to measure B-Pb with appropriate quality controls. The analyses were completed for 4285 women for lead. One sample had a lead level below the limit of detection: this was assigned a value of 0.7 times the lower limit of detection (limit of detection/√2) ^33, 34^. Of the 4285 mothers with a blood lead measurement during pregnancy, cases without a live birth and those who withdrew consent were excluded, leaving 4140 offspring for further analysis.

### Outcome measurements

The Children in Focus (CiF) cohort, comprising about 10% of the ALSPAC cohort, was selected from the last 6 months of ALSPAC births, occurring from June to December 1992. Mothers who had moved away from the study area, were lost to follow up, or whose baby had died, or who had two or more pregnancies in the study were excluded. Very premature babies (<33 weeks) participating in the Avon Premature Infant Project and their full-term controls were also excluded. 1432 children were invited to and attended at least one of a series of ten clinics at 4, 8, 12, 18, 25, 31, 37, 43, 49 and 61 months. Of the 1432 children, 574 had a prenatal B-Pb measurement. Clinic attendance rates are shown in *Extended data*, Supplementary Table 1 ^35^.

Measures of weight and length or height were taken at each clinic, as were head circumference and MUAC. Waist circumference was measured from 31 months onwards. The equipment used for each type of measurement is shown in *Extended data*, Supplementary Table 2 ^35^. BMI was derived as weight/height ^2^.

Anthropometric measures for BMI, weight and height were converted to z-scores using the British 1990 Growth Reference Data with adjustment for sex and age at clinic visit ^36, 37^.

### Covariates

We considered the following variables to be potential confounders of the relationship between prenatal lead exposure and offspring weight and height measures: maternal age (years), parity (n), education (None/Certificate of School Education/Vocational/O Level/A Level/Degree), pre-pregnancy height and weight (from which BMI (kg/m ^2^) was calculated), alcohol consumption in the first trimester (measures of alcohol per day) and cigarette smoking in the first trimester (n per day). These variables were derived from four postal self-completion questionnaires the mothers received during pregnancy ( http://www.bristol.ac.uk/alspac/researchers/questionnaires/; questionnaires #13548, 12452, 13194 and 12423).

### Statistical analysis

***Main analyses***. Analyses were carried out with IBM SPSS version 24 with the exception of z-scores, which were calculated in Stata (StataCorp, College Station, TX, USA) using the zanthro function.

We initially examined potential response bias in the sample by comparing the background characteristics of children enrolled in ALSPAC with a prenatal B-Pb measurement available to those without. We also compared the characteristics of children in ALSPAC-CiF who had a prenatal B-Pb measure with those without the measure. For all children with a prenatal B-Pb measure, we also compared the characteristics of those with complete data (exposure and all clinic measures) to those without complete data.

Multivariable linear regression modelling was used to examine the association of B-Pb with each outcome variable. B-Pb was log _10_-transformed to account for non-linearity in the variable. There were 137 cases with complete data on anthropometry and confounders: this was considered to have too little power to allow a complete case analysis. Analysis was therefore carried out on two datasets. (1) The first dataset was generated with multiple imputation by chained equation (MICE) applied to missing data for the 574 children with prenatal B-Pb included in ALSPAC-CiF. The patterns of missing data are shown in *Extended data*, Supplementary Table 3 ^35^. We generated 25 datasets with 200 iterations separating each imputed dataset. All outcomes and all confounders were imputed where missing (indicators used in the imputation were log _10_ prenatal B-Pb, birthweight, crown–heel length at birth, head circumference at birth and gestational age at birth). (2) The second dataset contained all available cases at each time point (n=275–374). The models for z-scores were adjusted for maternal age (years), maternal smoking (n per day), maternal education, parity, alcohol (n per day) and gestational age at sampling (weeks). Models for MUAC, waist circumference and head circumference were also adjusted for sex and age at clinic visit (months). As an additional analysis, the term sex×log _10_ B-Pb was added to the models to test for interactions. Where interaction tests showed evidence of associations (p≤0.2), further adjusted regression analyses were done with stratification by sex.

Regression diagnostics (primarily plots of residuals) were used to check that the models fitted the observed data well, to test the assumptions of regression, and to identify any cases that had undue influence on the model. Results are reported as adjusted unstandardised B coefficients with 95% confidence intervals at each clinic age.

***Sensitivity analysis***. Sensitivity analyses were conducted to verify the statistical model assumptions and test the robustness of the findings: the adjusted multivariate linear regression analyses were repeated after exclusion of children born preterm (livebirth <37 weeks) and/or low birthweight (livebirth <2500 g).

## Results

### Sample characteristics

The characteristics of the children with and without a prenatal B-Pb measurement in ALSPAC were similar except that children with prenatal B-Pb had a greater maternal age and higher maternal educational attainment ( Table 1). The characteristics of children with and without complete data in ALSPAC and ALSPAC-CiF are shown in *Extended data*, Supplementary Table 4 ^35^. Those with complete data in ALSPAC tended to include fewer mothers that smoked, mothers that were older and better educated, and fewer preterm babies than those with incomplete data. In ALSPAC-CiF, those with complete data tended to include mothers that were more likely to be older and better educated than those with incomplete data (data analyses were carried out on (1) an imputed ALSPAC-CiF database and (2) all available cases in ALSPAC-CiF).

**Table 1.** Characteristics of children enrolled in ALSPAC and in ALSPAC-CiF with or without a prenatal measurement of B-Pb.

	Participants in ALSPAC (n=14,602 live births)			Participants in ALSPAC-CiF (n=1432 attending at least one clinic)		
	Children with prenatal B-Pb measurement	Children without prenatal B-Pb measurement	P value	Children with prenatal B-Pb measurement	Children without prenatal B-Pb measurement	P value
**Mother**						
Pre-pregnancy BMI (kg/m2)						
Underweight (<18.5)	170 (4.8)	407 (5.1)	0.721	24 (4.6)	41 (5.5)	0.690
Normal weight (18.5–24.9)	2611 (74.3)	60008 (74.6)		377 (71.5)	533 (71.7)	
Overweight/obese (≥25.0)	735 (20.9)	1638 (20.3)		126 (23.9)	169 (22.7)	
Smoking						
No	3010 (79.2)	6713 (79.3)	0.884	458 (86.6)	651 (84.5)	0.308
Yes	792 (20.8)	1754 (20.7)		71 (13.4)	119 (15.5)	
Alcohol						
No	1880 (67.8)	2825 (68.4)	0.587	288 (66.5)	388 (65.0)	0.612
Yes	893 (32.2)	1304 (31.6)		145 (33.5)	209 (35.0)	
Parity						
0	1719 (44.4)	4083 (44.8)	0.704	255 (45.2)	378 (46.6)	0.609
≥1	21549 (55.6)	5030 (55.2)		309 (54.8)	433 (53.4)	
Age (years)						
≤25	1177 (28.4)	3134 (31.7)	<0.001	113 (19.5)	216 (25.4)	0.026
>25–30	1630 (39.4)	3886 (39.3)		249 (42.9)	350 (41.2)	
>30	1333 (32.2)	2874 (29.0)		218 (37.6)	283 (33.3)	
Education						
None/CSE/Vocational/O levels	2306 (62.0)	5743 (65.9)	<0.001	327 (57.9)	493 (61.8)	0.147
A level/degree	1416 (38.0)	1416 (34.1)		238 (42.1)	305 (38.2)	
**Child**						
Prenatal blood Pb	3.67±1.47 (range 0.20–19.14) µg/dl Median 3.40 (IQR 2.67-4.34) (n=4140)			3.59±1.50 (range 1.22–14.70) µg/dl Median 3.60 (IQR 2.61-4.16) (n=574)		
Sex						
Male	2146 (51.8)	5112 (51.7)	0.851	337 (58.1)	434 (51.1)	0.009
Female	1994 (48.2)	4783 (48.3)		243 (41.9)	415 (48.9)	
Preterm (livebirth <37 weeks)						
No	3882 (93.8)	9268 (93.7)	<816	559 (96.4)	796 (93.8)	0.028
Yes	258 (6.2)	637 (6.3)		21 (3.6)	53 (6.2)	
LBW (livebirth <2500 g)						
No	3867 (93.4)	9197 (92.9)	0.328	552 (95.2)	800 (94.2)	0.438
Yes	2273 (6.6)	698 (7.1)		28 (4.8)	49 (5.8)	

Pearson’s chi square test Values are n (%) LBW, low birth weight; BMI, body mass index

### Regression analyses

**Figure 1.**
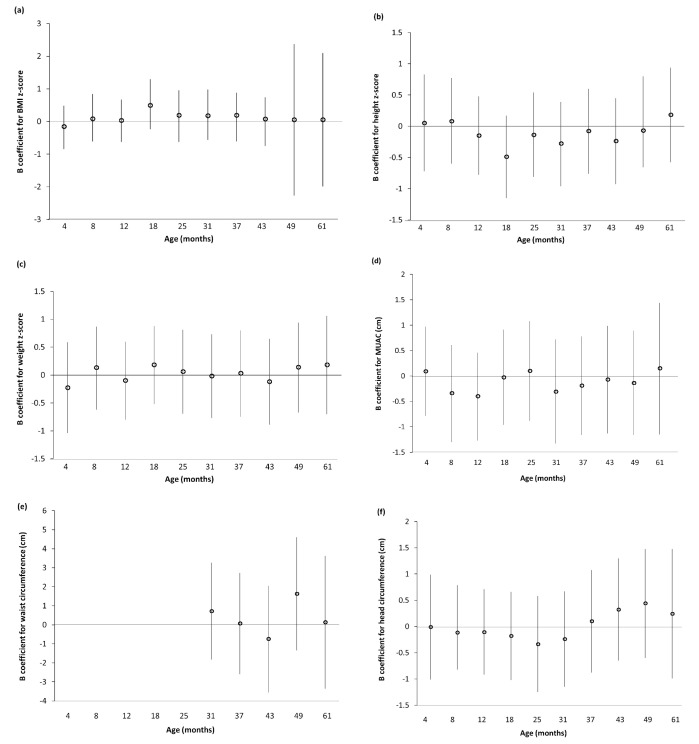
Association between prenatal log _10 _blood lead with z-scores for BMI, height and weight in children enrolled in ALSPAC attending the Children in Focus (CiF) clinics (multiple imputation, n=574). ( **a**) BMI z-score; ( **b**) height z-score; ( **c**) weight z-score; ( **d**) MUAC; ( **e**) waist circumference; ( **f**) head circumference. Values are unstandardised B coefficients with 95% confidence intervals from linear regression models. ( **a**)–( **c**) Adjusted for maternal age (years), maternal smoking (n per day), maternal education, parity (n), alcohol (n per day) and gestational age at sampling (weeks). ( **d**)–( **f**) Adjusted for maternal age (years), maternal smoking (n per day), maternal education, parity (n), alcohol (n per day), gestational age at sampling (weeks), maternal BMI (kg/m ^2^), sex and clinic age.

Anthropometric data at each age point are shown in *Extended data*, Supplementary Table 5 ^35^ for the imputed dataset and for all available cases. Z-scores for BMI, height and weight at each point are shown in *Extended data*, Supplementary Table 6. There were no associations between log _10_ B-Pb and z-scores for weight, height or BMI at any clinic age for the imputed dataset or all available cases ( Figure 1 and *Extended data*, Supplementary Figure 1 ^35^). Similarly, there were no associations between log _10_ B-Pb and MUAC, waist circumference or head circumference in either dataset ( Figure 1 and *Extended data*, Supplementary Figure 1 ^35^).

### Sex interactions

The p value for the association of the sex × log _10_ B-Pb interaction term was ≤0.2 for head circumference at 12, 31, 37, 43 and 61 months in the imputed dataset, but was >0.2 for all other outcomes ( *Extended data*, Supplementary Table 7 ^35^). On repetition of the adjusted regression analyses for head circumference after stratification by sex, B coefficients were consistently positive for girls and negative for boys (weak associations were evident for girls at 43 and 61 months, but not for boys). In all available cases, the p-value for the interaction was ≤0.2 for MUAC at 31 months, waist circumference at 37, 43, 49 and 61 months, and for head circumference at 4, 37, 43, 49 and 61 months ( *Extended data*, Supplementary Table 6 ^35^). On repetition of the adjusted regression analyses for head circumference after stratification by sex, associations were evident for head circumference at 43, 49 and 61 months in girls, but there were no associations in boys.

### Sensitivity analyses

Sensitivity analysis with exclusion of preterm and/or low birth weight participants showed very similar results to the data with the cases included: there were no associations between prenatal B-Pb and any of the anthropometric variables in adjusted analyses in either the imputed dataset or among all available cases ( *Extended data*, Supplementary Table 8 ^35^).

## Discussion

There was no evidence of any consistent associations between prenatal exposure to lead and a range of anthropometric variables from 4 to 61 months in this UK cohort of children. A previous study of prenatal lead exposure in ALSPAC showed associations with birthweight, crown–heel length and head circumference at birth ^38^: although the sample selection in the present study was different, our results suggest that if there were any small deficits apparent at birth, they were overcome in the first few months of life *ex utero*. This supports previous findings of a transient adverse effect of prenatal lead exposure providing exposure in childhood is low ^23, 29^. There was a weak positive association between prenatal B-Pb and head circumference in girls but not in boys (in whom the associations tended to be negative), but not for any other anthropometric measure. This suggests that boys may be more susceptible to the adverse effects of prenatal lead exposure than girls, although it is difficult to explain why this would affect head circumference alone.

Consistent with our findings, Gardner *et al*. ^20^ also found no associations of urine lead levels from 1505 mothers in Bangladesh with a range of growth variables measured from birth to 5 years of age including weight and height. Urine lead levels in the Bangladeshi mothers were not reported but may be relatively high as lead poisoning is a major public health problem in that country ^39^. Subsequent studies using cord blood lead concentrations rather than urine as the marker of exposure similarly found no relationship with stunting at 20–40 months of age in Bangladeshi children ^16^.

Several other studies have provided support for the traditional hypothesis of lead exposure being associated with decrements in growth. In the PROGRESS study in Mexico, where stunting and obesity are major public health problems, there were negative associations of third trimester B-Pb with height for age and weight for age in 4–6-year-old children, but no association with B-Pb in the second trimester ^27^. B-Pb in the second and third trimester (3.7±2.6 and 3.9±2.8 µg/dl, respectively) were very similar to that in the present study in the first trimester (3.59±1.50 µg/dl in ALSPAC-CiF). Other studies from South Korea and the USA have found negative associations of prenatal B-Pb with weight and height at 2 years old and at 1 year old, respectively, at moderate exposure concentrations (mean 1.25 and 2.8 µg/dl respectively) ^22, 25^. Liu *et al*. ^28^ also found negative associations between maternal patella (but not tibia) lead and BMI z-score, waist circumference, skinfolds thickness and body fat percentage in children in Mexico age 8 to 16 years.

Other studies, however, have suggested that lead exposure is positively associated with growth variables. A study in Korea including data from 280 children found positive associations of cord B-Pb with BMI z-scores from 18 to 27 months old, although not at earlier time intervals (cord B-Pb were relatively low: 1.39±0.9 µg/dl for boys and 1.21±0.7 µg/dl for girls). This was interpreted as providing support for a hypothesis that perinatal lead exposure is associated with childhood obesity, although the mechanism is unclear. It could perhaps be related to the developmental origins of health and disease hypothesis: if the fetus suffered stress *in utero* from lead exposure, which might restrict growth *in utero*, fetal programming might contribute to excess weight gain in early childhood if that stress was no longer present. However, cord B-Pb was associated with an increase rather than decrease in birth height in the Korean boys, but not in girls. These results are difficult to interpret and could be due to uncontrolled confounding or to chance. A similar longitudinal study in the USA including 1442 children with anthropometric variables from preschool age to adolescence also found that prenatal B-Pb was associated with the risk of the child being overweight or obese across the age span in a dose–response manner, with a suggestion of effect mediation by insulin and leptin concentrations ^17^. Using multilevel modelling of maternal perinatal blood bone lead concentrations to investigate the association with weight trajectories of about 1000 Mexican children from the ELEMENT cohort, Afeiche *et al*. ^19^ found that a 1 SD increase in patella lead was associated with 130 g decrement in weight at 5 years old in girls, but there was no association in boys. For tibia lead concentration, however, which was about 17% lower than patellar lead, there was no association with weight in girls. In boys, the association was positive at 36–60 months of age, with an 82.2 g increase in weight per 1 SD increase in tibia lead. The authors were not able to explain the difference in results from the two types of bone but noted that lead may accumulate more slowly in the tibia than the patella due to differences in vascularisation. Thus, the patella may reflect short-term exposure and the tibia longer term exposure. The mean B-Pb of these Mexican children across all ages (0–60 months) was 3.8±2.9 µg/dl (for comparison, B-Pb at 30 months old was similar at 4.2±3.1 µg/dl in a subsample of 582 children participating in ALSPAC ^6^). These findings of positive associations could reflect variation in the population prevalence of overweight and obesity, such that high background prevalence of overweight and obesity might mask adverse effects of prenatal B-Pb on anthropometric variables. However, overweight and obesity were prevalent both in children in the ALSPAC cohort ^40^ and in the Mexican cohort ^41^.

As the prenatal B-Pb in our study was relatively modest, it could be hypothesised that the exposure was too low to have any measurable effect on child growth. Thus, it would be expected that any adverse effects on growth would be magnified and be detectable at high exposure levels. However, in a study in Yugoslavia where participants living near a smelter had very high levels (20.6±7.38 µg/dl) compared with control areas (5.6±1.99 µg/dl), there were no associations with child BMI or height up to age 10 years ^26^. It may be that the effect of prenatal B-Pb may be moderated by subsequent exposure in early childhood: there was also a relatively high prenatal B-Pb of 7.5±1.6 µg/dl in the Cincinnati Lead Study in which associations of length in children between 3 and 33 months old were negative, but the effects were transient if subsequent child exposure was low ^23, 29^.

Overall, these studies do not serve as basis for strong comparisons or conclusions because of heterogeneity in the settings, and variations in participant numbers, exposure measures and levels, the outcomes measured, and the methods of data handling and statistical analyses. Even similar exposure measures, such as B-Pb, have resulted in conflicting findings, as discussed above ^22, 25– 27^). Maternal bone lead is thought to be a more stable marker of lead exposure in pregnancy than blood concentrations, but results again have been conflicting ^19, 27, 28^. The timing of exposure during pregnancy may be critical ^27^ and thus limit comparisons. The length of follow-up is also important as the emergence of an adverse effect may be missed if the study length is curtailed: with the exception of a study that followed children up to age of 10 years ^26^, most studies have included data up to the age of 5 or 6 years and some less than this ^22^.

There are several strengths of this study. First, the study involved a relatively large number of children with measures of prenatal B-Pb and frequent measures of growth from 4 to 61 months with age at clinic attendance recorded. This database includes a wide range of social and demographic information to enable the most appropriate selection of covariates. Second, cross-sectional analysis by linear regression of prenatal lead exposure and child growth can present statistical difficulties because of potential violations of the assumptions of independence and homogeneity and because of multicollinearity. There is the additional problem of ranges of actual age at anthropometric measurements. These difficulties were addressed in the present study by the use of z-scores, which standardises measurements across time ^42^, and multiple imputation. Cross-sectional analysis also fails to address the problem that the number of growth measurements may vary from child to child, and consequent deletion of missing cases may lead to bias and loss of power. This was again overcome in the present study with multiple imputation of data. Analysis of data from all available cases did not change the conclusions from the multivariable regression analyses with multiple imputed data. Third, the prenatal exposure was measured in the first half of pregnancy, providing confidence in the true fetal exposure levels in contrast to studies that have relied on cord blood levels or other matrices such as hair or urine.

There are also some limitations of the study. First, although we were able to account for many possible confounders in our analyses, we may have missed some important confounders while others may have been measured imperfectly. Second, effects on growth may have been too small, and therefore clinically unimportant, to have been detected with the methods for anthropometric measures. Third, the role of concomitant exposure to other pollutants and other factors that may be associated with growth might have the effect of masking associations (for example, polychlorinated biphenyls, 2.5 µm particulate matter). These measures were not included and should be explored further in other studies. Fourth, the time lapse between the exposure and the outcomes in this study means that the child will have experienced unknown further exposure to lead postnatally that was not accounted for.

## Conclusion

There was no consistent evidence of associations between prenatal exposure to lead and growth and weight from 4 to 61 months in this large cohort of children in the UK. There was evidence for a weak positive effect of prenatal lead exposure on head circumference in girls at 61 months: this may have been due to confounding that we were unable to account for. Overall, the study provides no evidence for an adverse effect of prenatal lead exposure on preschool growth in this population.

## Data availability

### Underlying data

ALSPAC data access is through a system of managed open access. The steps below highlight how to apply for access to the data included in this research article and all other ALSPAC data. The datasets presented in this article are linked to ALSPAC project number B2116; please quote this project number during your application. The ALSPAC variable codes highlighted in the dataset descriptions can be used to specify required variables.

- 1. Please read the ALSPAC access policy ( https://www.bristol.ac.uk/media-library/sites/alspac/documents/researchers/data-access/ALSPAC_Access_Policy.pdf) which describes the process of accessing the data and samples in detail, and outlines the costs associated with doing so.
- 2. You may also find it useful to browse our fully searchable research proposals database ( https://proposals.epi.bristol.ac.uk/), which lists all research projects that have been approved since April 2011.
- 3. Please submit your research proposal for consideration by the ALSPAC Executive Committee. You will receive a response within 10 working days to advise you whether your proposal has been approved.

If you have any questions about accessing data, please email alspac-data@bristol.ac.uk.

The ALSPAC data management plan describes in detail the policy regarding data sharing, which is through a system of managed open access. The study website also contains details of all the data that is available through a fully searchable data dictionary: http://www.bristol.ac.uk/alspac/researchers/data-access/data-dictionary/.

### Extended data

Figshare: Prenatal lead exposure: associations with growth and anthropometry in early childhood in a UK observational birth cohort study: Supplementary material. https://doi.org/10.6084/m9.figshare.14101976 ^35^.

This file contains the following extended data:

- **Supplementary Figure 1.** Association between prenatal log _10_ blood lead with z-scores for BMI, height and weight in children enrolled in ALSPAC attending the Children in Focus (CiF) clinics (all available cases).
- **Supplementary Table 1.** Number of children enrolled in ALSPAC-CiF clinics who also had a prenatal measure of blood lead and confounder data.
- **Supplementary Table 2.** Equipment used for anthropometric measurements for ALSPAC-CiF clinics.
- **Supplementary Table 3.** Fractions of missing data in ALSPAC-CiF sample (attended at least one clinic and with prenatal B-Pb measure) (n=574).
- **Supplementary Table 4.** Characteristics of children enrolled in ALSPAC-CiF subsample with complete data on prenatal measurement of B-Pb, confounders and clinic data versus those with data on prenatal measurement of B-Pb but incomplete data on confounders and clinic data.
- **Supplementary Table 5.** Anthropometry data at each time point in ALSPAC-CiF participants with prenatal B-Pb measure (multiple imputation and all available cases).
- **Supplementary Table 6.** Z-scores for anthropometry data at each time point in ALSPAC-CiF participants with prenatal B-Pb measure (multiple imputation, n=574).
- **Supplementary Table 7.** Interaction between prenatal B-Pb and sex in the associations between prenatal log _10_ blood lead with MUAC, waist circumference and head circumference in children attending ALSPAC-CiF clinics (multiple imputation and all available cases), with the associations for head circumference further stratified by sex.
- **Supplementary Table 8.** Sensitivity analysis for associations between prenatal log _10_ blood lead and anthropometric variables in children attending ALSPAC-CiF clinics (multiple imputation and all available cases) (low birthweight and/or preterm children excluded).

Extended data are available under the terms of the Creative Commons Attribution 4.0 International license (CC-BY 4.0).
