# Supplementary material for: Prenatal lead exposure: associations with growth and anthropometry in early childhood in a UK observational birth cohort study
Source: Wellcome Open Res. 2026 Mar 12;5:235. Originally published 2020 Oct 8. [Version 3] doi: 10.12688/wellcomeopenres.16338.3 (PMC13062770; doi:10.12688/wellcomeopenres.16338.3)
Supplement: Peer review history

# Reviewer report: Reviewer response for version 3

**DOI:** 10.21956/wellcomeopenres.28768.r151087

**Published:** 2026-04-11

**Authors:** Maria Kippler

**Affiliations:** 1 Institute of Environmental Medicine, Karolinska Institutet, Stockholm, Sweden

The overarching aim of the present study was to examine associations of prenatal exposure to lead on children’s growth from 4 to 61 months of age. This was investigated in 574 mother-child dyads from the well-established UK Avon Longitudinal Study of Parents and Children (ALSPAC). Lead exposure was determined by measurement in maternal blood in the first trimester. Several anthropometric parameters were measured repeatedly (total of 10 occasions). This study adds important knowledge to a research area which has gained limited attention and where data is still conflicting. The study is thoroughly evaluated and nicely presented. However, some minor clarifications could make it even more readily available and interesting for the reader.

 Introduction: It is stated that: The potential mechanisms for the adverse effect of prenatal lead exposure on growth are largely undefined but may include direct interference with bone cell function and/or disruption of hormones (such as vitamin D and thyroid hormones) that are involved in the synthesis of the bone matrix, as well as epigenetic mechanisms. I fully agree with these hypotheses, however, it would be beneficial to also add some supporting references.

 Exposure measurement: It is very informative that time frame of sample collection is presented in detail (median of 11 weeks’ gestation (IQR 9–13 weeks) with a mode of 10 weeks). However, the meaning of a mode of 10 weeks is not clear. Could this be clarified?

 Exposure measurements: Please add the limit of detection along with the statement that one sample was assigned a value of 0.7 times the lower limit of detection (limit of detection/√2).

 Outcome assessments: It is stated that BMI was derived as weight/height and then there is reference afterwards (numbered 2, which does not seem to belong here). Could this be a typo?

 Statistical analysis: Multivariable linear regression modelling was used to examine the association of B-Pb with each outcome variable. Did the authors also consider using mixed linear regression modelling, which seems to be a very good fit for the present data, especially since there is missing data at several time points (which is to be expected during such extensive data collection).

 Statistical analysis: Regarding the selection of covariates, could some justification be added regarding the final selection? Or potentially why some stated factors like maternal weight and height were not included.

 In the end of the first section of the Discussion, the authors initially present the positive association between prenatal lead exposure and head circumference, which was only apparent in girls, while there was a tendency of a negative trend in boys. Thereafter, it is suggested that boys are more susceptible than girls. This is interesting, although not straightforward when it comes to interpretation, which needs to be acknowledged and discussed further. Therefore, maybe the last statement should be revised so that it also coincides with the conclusion at the end of the discussion.  

 Discussion: When discussing different exposure biomarkers and stating that timing of exposure measurements is critical, it would have been very interesting to expand on what is known about timing of exposure during pregnancy as blood lead levels tend to change during pregnancy or is data still too limited to say anything about this?

Is the work clearly and accurately presented and does it cite the current literature?

Yes

If applicable, is the statistical analysis and its interpretation appropriate?

Partly

Are all the source data underlying the results available to ensure full reproducibility?

Yes

Is the study design appropriate and is the work technically sound?

Yes

Are the conclusions drawn adequately supported by the results?

Yes

Are sufficient details of methods and analysis provided to allow replication by others?

Partly

Reviewer Expertise:

Early-life exposure to environmental pollutants, in particular metals and other trace elements, and their impact on child health and development.

I confirm that I have read this submission and believe that I have an appropriate level of expertise to confirm that it is of an acceptable scientific standard, however I have significant reservations, as outlined above.

# Reviewer report: Reviewer response for version 3

**DOI:** 10.21956/wellcomeopenres.28768.r152706

**Published:** 2026-04-08

**Authors:** Pam Factor-Litvak

**Affiliations:** 1 Columbia University,, New York, USA

This is a generally well-done paper in an exemplary birth cohort which examines associations between prenatal exposure to lead (as blood lead concentrations, BPb) and childhood growth.  As do many of the previous studies, the paper does not find appreciable associations between exposure and weight, height, BMI, head circumference, waist circumference or mid upper arm circumference.  

 I have a suggestion for an additional analysis and some suggestions about the interpretation of the sex-specific results.  Also, one point of clarification: bone lead measures during pregnancy may not be a good marker of fetal exposure as maternal bone is remodeled during pregnancy, leading to higher levels in the blood, which is more available to the fetus.   

 Analysis: the paper would be stronger if the outcome variables were, in a subsequent analysis, analyzed as trajectories and then the interpretation would be based on the rate of growth in relation to prenatal lead exposure.

 Interpretation: Although not significant at any follow up time point, there is a remarkable consistency that the relationship between lead and head circumference in girls is positive and that in boys is negative.  The authors point this out but I think it is strongly suggestive and perhaps not significant due to sample size considerations.  If this is true, however, then it would be similar to the associations between lead and neurodevelopmental outcomes which is more pronounced in boys than girls.

Is the work clearly and accurately presented and does it cite the current literature?

Yes

If applicable, is the statistical analysis and its interpretation appropriate?

Partly

Are all the source data underlying the results available to ensure full reproducibility?

Yes

Is the study design appropriate and is the work technically sound?

Yes

Are the conclusions drawn adequately supported by the results?

Partly

Are sufficient details of methods and analysis provided to allow replication by others?

Yes

Reviewer Expertise:

Early life environmental and social exposures and outcomes over the life course; epidemiologic methods; epidemiology of neurological disorders

I confirm that I have read this submission and believe that I have an appropriate level of expertise to confirm that it is of an acceptable scientific standard, however I have significant reservations, as outlined above.

# Reviewer report: Reviewer response for version 2

**DOI:** 10.21956/wellcomeopenres.18406.r42985

**Published:** 2021-03-25

**Authors:** Manish Arora

**Affiliations:** 1 Department of Environmental Medicine and Public Health, Icahn School of Medicine at Mount Sinai, New York City, NY, USA

The authors have adequately addressed my comments. I support the indexing of this manuscript. Thank you for the opportunity to review.

Is the work clearly and accurately presented and does it cite the current literature?

Yes

If applicable, is the statistical analysis and its interpretation appropriate?

Partly

Are all the source data underlying the results available to ensure full reproducibility?

Yes

Is the study design appropriate and is the work technically sound?

Yes

Are the conclusions drawn adequately supported by the results?

Yes

Are sufficient details of methods and analysis provided to allow replication by others?

Yes

Reviewer Expertise:

NA

I confirm that I have read this submission and believe that I have an appropriate level of expertise to confirm that it is of an acceptable scientific standard.

# Reviewer report: Reviewer response for version 2

**DOI:** 10.21956/wellcomeopenres.18406.r42986

**Published:** 2021-03-10

**Authors:** Caroline H. D. Fall

**Affiliations:** 1 Medical Research Council Lifecourse Epidemiology Unit, University of Southampton, Southampton, UK

The authors have addressed most of my points. However the B coefficient information in the abstract is still incomplete and is therefore uninterpretable - a regression coefficient should state the amount by which the outcome changes (in this case 1.59 cm) per unit of lead exposure (the unit of lead exposure is missing).

Is the work clearly and accurately presented and does it cite the current literature?

Yes

If applicable, is the statistical analysis and its interpretation appropriate?

Yes

Are all the source data underlying the results available to ensure full reproducibility?

Yes

Is the study design appropriate and is the work technically sound?

Yes

Are the conclusions drawn adequately supported by the results?

Yes

Are sufficient details of methods and analysis provided to allow replication by others?

Yes

Reviewer Expertise:

Epidemiology

I confirm that I have read this submission and believe that I have an appropriate level of expertise to confirm that it is of an acceptable scientific standard, however I have significant reservations, as outlined above.

# Response

**Published:** 2026-02-18

**Authors:** Caroline Taylor

**Affiliations:** Bristol Medical School, University of Bristol, Bristol, UK

The unit for the B coefficient has been added to the abstract (and also in the legend for Figure 1).

# Reviewer report: Reviewer response for version 1

**DOI:** 10.21956/wellcomeopenres.17963.r42204

**Published:** 2021-02-09

**Authors:** Caroline H. D. Fall

**Affiliations:** 1 Medical Research Council Lifecourse Epidemiology Unit, University of Southampton, Southampton, UK

This well-written paper reports an analysis in the ALSPAC cohort of children's anthropometry between the ages of 4 and 61 months in relation to pre-natal lead exposure, assessed from maternal 1st trimester blood lead levels. There was no evidence that higher maternal blood lead concentrations were associated with any growth deficit in the children. Among girls, there was weak evidence that greater lead exposure was associated with larger head circumference between the ages of 3 and 5 years.

 The data have been well analysed and presented. The authors have described clearly the fall-off in numbers from women enrolled to blood sampling in pregnancy to live births to the children in focus (CiF) sub-sample to clinic follow-up, and made extensive efforts to assess representativeness, and to maximise the use of the data while checking for any bias. 

 It must have been a dilemma whether or not to report the sex interactions and the positive association between lead exposure and head circumference in girls, which may well be a chance finding. There are statistically significant sex interactions, though probably not many more than would be expected by chance.  On the other hand, the difference in the direction of associations between boys and girls is impressive in size and consistent. Given that there is a literature linking pre-natal lead exposure to larger body size in children, albeit inconsistent in terms of both the measurement and the sex concerned, it would have been wrong not to present this data, and I think the authors have been balanced in their presentation and conclusions on this point. 

 Why are lead levels reported as mean (SD) in the abstract and Table 1 when the variable was skewed? - wouldn't median (IQR) be more appropriate?

 The B coefficient in the abstract is incomplete and needs clarifying. It is given in cm. Please clarify the units of exposure for this analysis.

 Supplementary table 5: Please provide the SDs for the children's anthropometry (imputed data) and also add to this table (or include a separate table) the Z-scores for height, weight and BMI according to the UK growth reference at each age.

Is the work clearly and accurately presented and does it cite the current literature?

Yes

If applicable, is the statistical analysis and its interpretation appropriate?

Yes

Are all the source data underlying the results available to ensure full reproducibility?

Yes

Is the study design appropriate and is the work technically sound?

Yes

Are the conclusions drawn adequately supported by the results?

Yes

Are sufficient details of methods and analysis provided to allow replication by others?

Yes

Reviewer Expertise:

Epidemiology

I confirm that I have read this submission and believe that I have an appropriate level of expertise to confirm that it is of an acceptable scientific standard, however I have significant reservations, as outlined above.

# Response

**Published:** 2021-02-24

**Authors:** Caroline Taylor

**Affiliations:** Bristol Medical School, University of Bristol, Bristol, UK

1. Why are lead levels reported as mean (SD) in the abstract and Table 1 when the variable was skewed? - wouldn't median (IQR) be more appropriate?

The median and IQR values are now reported in the abstract and Table 1.

  

1. The B coefficient in the abstract is incomplete and needs clarifying. It is given in cm. Please clarify the units of exposure for this analysis.

The data for the head circumference measurements are in cm. Here we state the unstandardised B coefficient (rather than beta the standardised coefficient in SD units) so cm is the correct unit.

  

1. Supplementary table 5: Please provide the SDs for the children's anthropometry (imputed data) and also add to this table (or include a separate table) the Z-scores for height, weight and BMI according to the UK growth reference at each age.

Thank you for noticing that the SDs had been omitted – they have now been included.

 A new supplementary table 6 has been added to show the z-scores for the imputed database and the remaining supplementary tables re-numbered.

# Reviewer report: Reviewer response for version 1

**DOI:** 10.21956/wellcomeopenres.17963.r40806

**Published:** 2020-11-10

**Authors:** Manish Arora

**Affiliations:** 1 Department of Environmental Medicine and Public Health, Icahn School of Medicine at Mount Sinai, New York City, NY, USA

The authors have used a well-established cohort - UK Avon Longitudinal Study of Parents and Children (ALSPAC) - to undertake an analysis of pregnancy measures of maternal blood lead (Pb) and birth and childhood growth measures. The lack of any significant findings do not diminish the importance of the work, as these negative findings are of value to the body of literature on the adverse impacts of Pb. However, the main concern is in the underlying concept that Pb alone is acting to impact health - an increasing body of literature now supports the role of lead in a mixture context.

 There are two main recommendations to improve the manuscript:

1. There is a missed opportunity to place Pb exposure in the context of the multiple exposures that the ALSPAC participants have experienced, and in many cases data are available is my understanding. In essence, a mixtures analysis would be much more in keeping with the state of the science. Although I do not do work in ALSPAC, I am aware of publications on air-pollution and other environmental factors. Consider what other environmental exposures are measured in ALSPAC?
2. Related to the earlier point, the few other environmental and dietary exposures that are included in the analysis are treated as co-variates - again there is a missed opportunity to do mixtures analysis and use more advanced techniques such as a weighted exposure index-type approach that would allow the impact of Pb as part of an exposure mixture to become apparent.
3. Authors should not refer to this level of exposure as "low". The levels reported are not "low" compared to current norms and known health effects.

Is the work clearly and accurately presented and does it cite the current literature?

Yes

If applicable, is the statistical analysis and its interpretation appropriate?

Partly

Are all the source data underlying the results available to ensure full reproducibility?

Yes

Is the study design appropriate and is the work technically sound?

Yes

Are the conclusions drawn adequately supported by the results?

Yes

Are sufficient details of methods and analysis provided to allow replication by others?

Yes

Reviewer Expertise:

environmental epidemiology, exposure biology

I confirm that I have read this submission and believe that I have an appropriate level of expertise to confirm that it is of an acceptable scientific standard, however I have significant reservations, as outlined above.

# Response

**Published:** 2021-02-24

**Authors:** Caroline Taylor

**Affiliations:** Bristol Medical School, University of Bristol, Bristol, UK

1. There is a missed opportunity to place Pb exposure in the context of the multiple exposures that the ALSPAC participants have experienced, and in many cases data are available is my understanding. In essence, a mixtures analysis would be much more in keeping with the state of the science. Although I do not do work in ALSPAC, I am aware of publications on air-pollution and other environmental factors. Consider what other environmental exposures are measured in ALSPAC?
2. Related to the earlier point, the few other environmental and dietary exposures that are included in the analysis are treated as co-variates - again there is a missed opportunity to do mixtures analysis and use more advanced techniques such as a weighted exposure index-type approach that would allow the impact of Pb as part of an exposure mixture to become apparent.

Response: Thank you for these two related comments. We agree that exposures to pollutants and other environmental factors do not usually occur in isolation and that a weighted exposure approach would be of interest. Our aim here was to look specifically at lead exposure, which is very under-researched in the UK. This addresses a different question to the one of mixture analysis, which would address whether additional metals in combination with lead affect growth in a more potent way. We have recently curated environmental data available in ALSPAC as a resource (Boyd et al. 2019) and this could be used as the basis for a further analysis as you suggest.

1. Authors should not refer to this level of exposure as "low". The levels reported are not "low" compared to current norms and known health effects.

Response: The descriptor ‘low level’ in reference to the exposure to lead has been removed throughout.
